# Forkhead Box M1 positively regulates UBE2C and protects glioma cells from autophagic death

**DOI:** 10.1080/15384101.2017.1356507

**Published:** 2017-08-02

**Authors:** Liang Guo, Zhiming Ding, Nunu Huang, Zhengsong Huang, Nu Zhang, Zhibo Xia

**Affiliations:** Department of Neurosurgery, The First Affiliated Hospital of Sun Yat-sen University, Guangzhou, Guangdong Province, China

**Keywords:** autophagy, FoxM1, glioma, transcription, UBE2C

## Abstract

Ubiquitin-conjugating enzyme E2C (UBE2C) is characterized as a crucial molecule in cancer cell growth that plays an essential role in the development of gliomas, but the detailed mechanisms have not been fully elucidated. In this study, we found that Forkhead box transcription factor M1 (FoxM1) overexpression increased UBE2C expression, whereas FoxM1 suppression inhibited UBE2C expression in glioma cells. In addition, high FoxM1/UBE2C expression was significantly correlated with poor prognosis in glioma. We subsequently demonstrated that UBE2C was a direct transcriptional target of FoxM1, and site-directed mutations markedly down-regulated UBE2C promoter activity. Moreover, UBE2C siRNA (si-UBE2C) significantly induced glioma cell autophagy and increased both mCherry-LC3 punctate fluorescence and LC3B-II/LC3-I expression. Notably, the si-UBE2C-induced decrease in cell viability was markedly inhibited by the autophagy inhibitor bafilomycin A1. The silencing of UBE2C resulted in a distinct inhibition of the PI3K-Akt-mTOR pathway, which functions in the negative modulation of autophagy. Collectively, our findings provide clinical and molecular evidence that FoxM1 promotes glioma progression by enhancing UBE2C transcription and that the inhibition of UBE2C partially induces autophagic glioma cell death. Thus, targeting the FoxM1-UBE2C axis has therapeutic potential in the treatment of gliomas.

## Introduction

Glioma is a highly invasive malignant tumor type found in the central nervous system and is among the most aggressive and challenging neoplasms to treat.^1^ Although multimodal treatments involving surgical resection followed by chemotherapy or radiotherapy have been performed in the clinic, gliomas remain highly resistant to these therapies.^2^ Glioblastoma is known as the most malignant type of glioma and is characterized by extremely rapid progression and a poor prognosis, with a mean survival time of only 12–14 months after surgical resection.^3^^,^^4^ Unfortunately, to date, little is known about the reasons for the deterioration and poor prognosis for gliomas. Therefore, it is imperative to explore the underlying oncogenic molecular mechanisms.

Forkhead box transcription factor M1 (FoxM1) is a typical proliferation-specific transcription factor, which is up-regulated in various types of human malignancies, including lung cancer, hepatoma, prostate cancer, breast cancer, sarcoma, pancreatic cancer and glioma.^5-11^ FoxM1 regulates the transition from the G1 to S and the G2 to M phase in mitosis,^12^ and dysregulated FoxM1 expression results in cell cycle arrest and chromosome mis-segregation in tumor cells.^13^ In our previous studies of the oncogenic roles of this molecule, we found that FoxM1 up-regulation increased and FoxM1 down-regulation inhibited angiogenesis in glioma cells.^14^ We further revealed that high FoxM1 expression enhanced the tumorigenicity of glioma cells.^15^ Moreover, several other previous studies have also reported that enhanced levels of FoxM1 led to cancer cell migration, invasion and metastasis via regulating signal pathways or inducing the epithelial-to-mesenchymal transition.^16-18^

Clinicopathological investigations have proposed an up-regulation of ubiquitin-conjugating enzyme E2C (UBE2C) in gliomas. UBE2C is a member of the E2 gene family and codes for a 19.6 kDa protein involved in ubiquitination-dependent proteolysis.^19^^,^^20^ UBE2C was revealed to be implicated in intracellular protein degradation via the mitotic spindle assembly checkpoint pathway.^21^ Mounting evidence indicates that UBE2C is vital in many biological processes, such as carcinogenesis,^22^ cell proliferation,^23^ the cell cycle^24^ and apoptosis.^25^ Donato et al.^26^ revealed a remarkable association between UBE2C expression and the histological grade of gliomas. Thereafter, Jiang et al.^27^^,^^28^ analyzed the UBE2C expression levels in gliomas of different grades and further demonstrated that UBE2C knockdown inhibits glioma cell proliferation and enhances apoptosis. However, the detailed molecular mechanism by which UBE2C contributes to malignant glioma (MG) progression remains undefined.

Previously, many studies have identified that some anti-cancer agents, such as arsenic trioxide, rapamycin and concanavalin A,^29-31^ induce cell death with autophagic features in various tumors. Autophagy, an evolutionarily conserved defense mechanism, is closely involved in the maintenance of homeostasis, typically via degrading damaged proteins or organelles. Despite its bidirectional contribution to cell healing and cell death, autophagy has recently received more attention as an optimal surveillance mechanism for tumor suppression among numerous therapeutic interventions. However, the association between autophagy and glioma progression is poorly characterized.

In the present study, we initially provided evidence that FoxM1 triggered UBE2C up-regulation by directly binding to the UBE2C promoter regions and facilitated its expression at the transcriptional level. Moreover, our findings indicated another type of programmed cell death aside from apoptosis and elaborated upon the process of autophagy induced by si-UBE2C in glioma cells. These findings provide new insights into the regulatory mechanisms of FoxM1 as well as the autophagic function of UBE2C in gliomas.

## Results

### UBE2C overexpression and its correlation with FoxM1 in human gliomas

**Figure 1.**
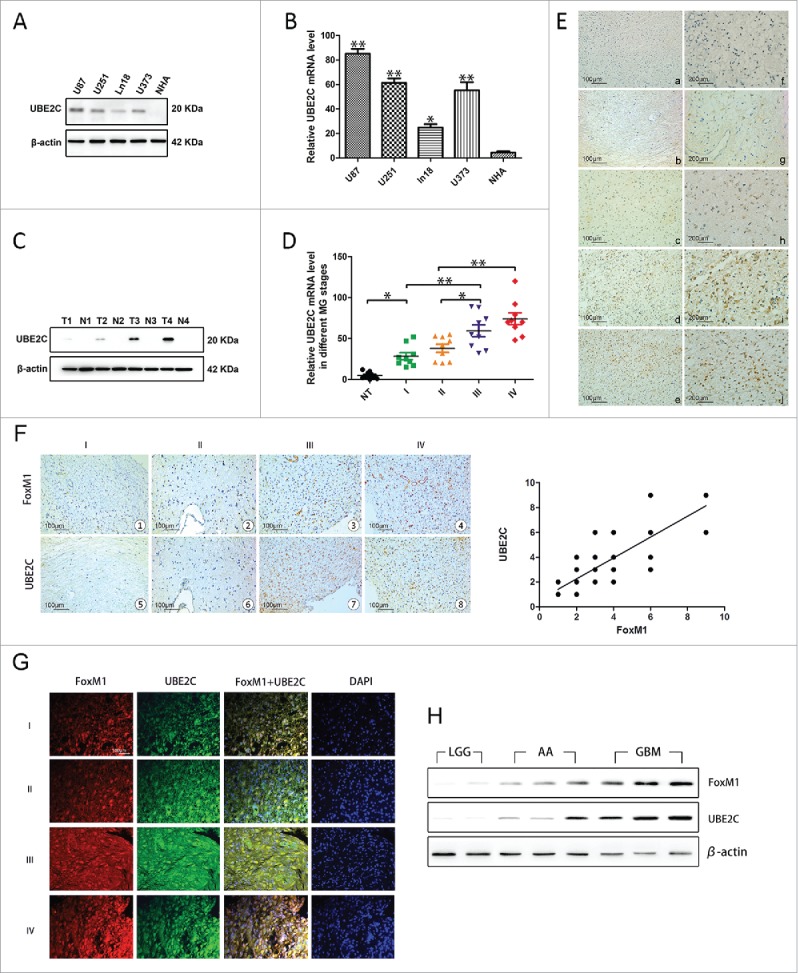
UBE2C overexpression and its correlation with FoxM1 in human gliomas. (A) The Western blot analysis showed varied UBE2C expression in MG U87, U251, Ln18, and U373 cell lines, whereas no UBE2C protein was expressed in NHAs. (B) The qRT-PCR results show the relative UBE2C mRNA expression levels in the above glioma cell lines; very little UBE2C mRNA expression was observed in NHAs; *p < 0.05, **p < 0.01 vs. NHAs. (C) Western blot analysis results of gliomas of different WHO grades, where T1-T4 refer to grades I to IV, and N indicates adjacent normal brain tissues. (D) The UBE2C mRNA expression levels in MG tissues of different stages; normal brain tissues were used as a control; *p < 0.01, **p < 0.001. (E) IHC with UBE2C antibodies on NHB and MG tissues. Magnification × 200: (a) NHB tissue; (b) pilocytic astrocytoma (WHO grade I); (c) diffuse astrocytoma (WHO grade II); (d) aplastic astrocytoma (WHO grade III); (e) GBM (WHO grade IV); (f-j) magnification × 400 of the corresponding pictures on the left. (F) Upper panel, FoxM1 expression was examined by IHC in 154 glioma specimens of different WHO grades and in 27 normal tissues. Representative images are shown. Lower panel, the level of UBE2C staining in the adjacent slices. Magnification x200 (r = 0.789, p < 0.001). (G) Immunofluorescence assay showing the colocalization of FoxM1 with UBE2C in 154 specimens. Representative images are shown. Scale bar, 200 µm. (H) FoxM1 and UBE2C protein expression levels were determined by Western blot analysis with protein extracts from 2 LGG, 3 AA and 3 GBM frozen tissue samples.

First, U87-MG, U251, Ln18 and U373 cells were used in our study to examine the expression of UBE2C in glioma cell lines. As indicated in Fig. 1A and B, variable UBE2C expression levels were observed at both the mRNA and protein levels in the diverse glioma cell lines, whereas there was almost no UBE2C expression in NHAs. Moreover, as shown in Fig. 1C and D, UBE2C mRNA levels were significantly up-regulated with advancing glioma stage, as determined by qRT-PCR, and the protein expression exhibited a similar trend, as stage IV showed the highest protein levels.

Next, we investigated UBE2C expression in 154 glioma tissues of different grades according to the WHO classification and 27 normal human brain (NHB) tissues. The immunohistochemical staining revealed that all the NHB tissues displayed no or extremely low levels of UBE2C protein expression (IRS ≦2, 27/27); however, 63% of the glioma tissues (97/154) exhibited strong UBE2C immunoreactivity (IRS ≧4). Furthermore, according to the immunohistochemistry (IHC) results, we found that UBE2C expression might correlate with the pathological stage of glioma patients because the IRS of UBE2C increased with increasing stage; stage I had the lowest UBE2C IRS, and stage IV had the highest, indicating a crucial role of UBE2C expression in the pathogenesis of glioma (Fig. 1E). Moreover, to confirm the clinical and pathological relevance of the Western blot, qRT-PCR and IHC results, we performed a clinicopathological analysis of UBE2C in 154 glioma cases (Table S1).

In addition, we determined FoxM1 expression in the same series of glioma specimens; a pattern similar to that of UBE2C expression was observed (Fig. 1F). We further analyzed the IHC scores and found a significant correlation between the expression levels of UBE2C and FoxM1 (Fig. 1F, r = 0.789, p < 0.001). The colocalization of UBE2C and FoxM1 expression was confirmed by a double immunofluorescence assay, showing that the expression levels of both UBE2C and FoxM1 were correlated with the glioma histological grade (Fig. 1G). Furthermore, a Western blot analysis was conducted using total protein extracts from frozen human glioma tissues of 2 low-grade glioma (LGG), 3 anaplastic astrocytoma (AA) and 3 glioblastoma multiforme (GBM) samples. As shown in Fig. 1H, UBE2C expression was positively associated with FoxM1 expression.

**Figure 2.**
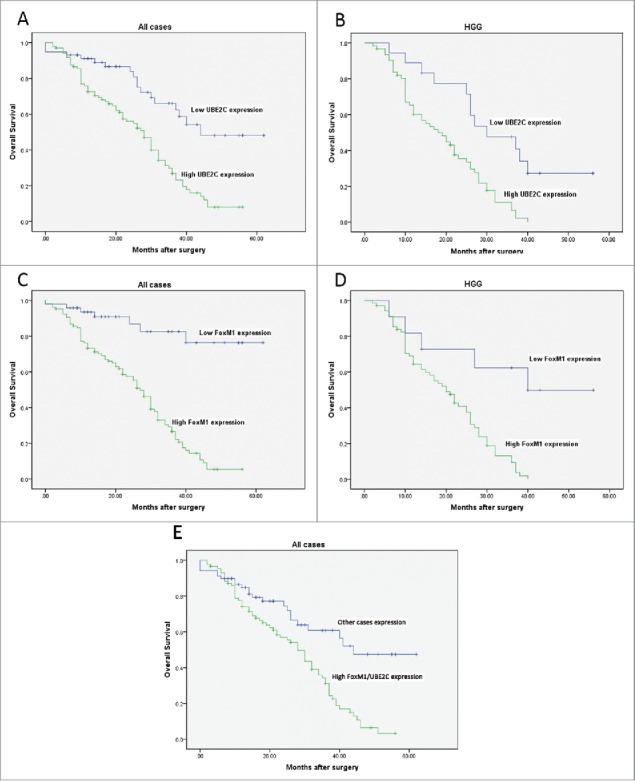
Kaplan–Meier curves for overall survival with gliomas. (A-B) Kaplan–Meier curves for OS in patients with gliomas divided according to UBE2C expression levels among all cases and high-grade gliomas (WHO grades III and IV). (C-D) Kaplan–Meier curves for OS in patients with gliomas divided according to FoxM1 expression levels among all cases and high-grade gliomas. (E) Kaplan–Meier curves for the OS of patients with gliomas with high FoxM1/UBE2C expression levels and of other patients.

In the follow-up period, we collected clinical data from the glioma patients. Overall, 89 of the 154 patients died (18 from the low UBE2C expression group and 71 from the high UBE2C expression group; 7 from low FoxM1 expression group and 82 from the high FoxM1 expression group). The Kaplan–Meier curve analysis showed that the survival rate in cases with high UBE2C expression was significantly lower than that in cases with low UBE2C expression (p = 0.001). The mean survival time in cases with low UBE2C expression was 43.63 ± 3.27 months (95% CI, 37.22–50.04), whereas the mean survival time in cases with high UBE2C expression was 26.58 ± 1.65 months (95% CI, 23.35–29.81, p < 0.01, Fig. 2A). We classified the cases into two groups: the high-grade group (WHO grades III–IV) and the low-grade group (WHO grades I–II). Likewise, the survival rate in the high-grade group was significantly lower than that in the low-grade group (p < 0.01). Moreover, in the high-grade group, the survival rate of the 18 patients with low UBE2C expression was significantly higher than that of the 62 patients with high UBE2C expression (p < 0.01, Fig. 2B).

Simultaneously, the survival rate in cases with high FoxM1 expression was remarkably lower than that in cases with low FoxM1 expression (p < 0.01). The mean survival time in cases with low FoxM1 expression was 52.67 ± 3.1 months (95% CI, 46.42–58.91), whereas the mean survival time of cases with high FoxM1 expression was 26.00 ± 1.50 months (95% CI, 23.07–28.94, p < 0.01, Fig. 2C). Likewise, in the high-grade group, the survival rate of the 11 patients with low FoxM1 expression was significantly higher than that of the 69 patients with high FoxM1 expression (p < 0.01, Fig. 2D).

Furthermore, the survival rate of patients with both high FoxM1/UBE2C expression levels was markedly lower than that in the other patients (p < 0.01, Fig. 2E). The mean survival time of patients with high FoxM1/UBE2C expression was 25.17 ± 2.40 months (95% CI, 23.29–28.71, p < 0.01), whereas the mean survival time of the other patients was 44.01 ± 3.12 months (95% CI, 39.17–50.34).

### Bioinformatics analysis of UBE2C

**Figure 3.**
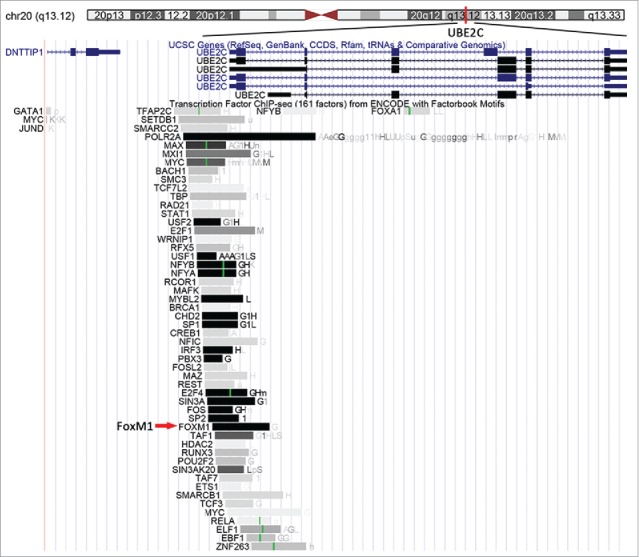
Predicted TFs, including FoxM1, are predicted to bind the UBE2C promoter region. The location of the UBE2C gene in the human genome is highlighted by the red bar. The FoxM1 transcription factor is indicated by the red arrow.

A bioinformatics analysis of the UBE2C gene using the University of California Santa Cruz (UCSC) Genome Browser (http://genome.ucsc.edu) indicated that UBE2C is located at 20q13.12, and many transcription factors (TFs), including FoxM1, were also listed (Fig. 3). We searched the factorbook transcription factor ChIP-Seq (161 factors) dataset from ENCODE for FoxM1 and found that the cluster score (out of 1000) was 1000, suggesting that FoxM1 might bind to the UBE2C promoter region.

### Silencing FoxM1 inhibited UBE2C expression in glioma cell lines

**Figure 4.**
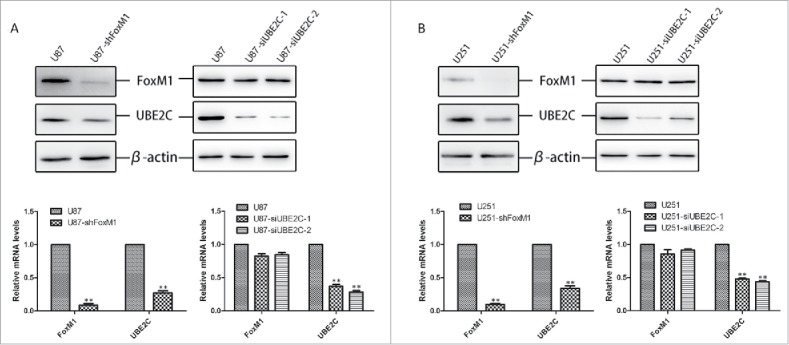
Silencing FoxM1 inhibits UBE2C expression in glioma cell lines. (A) Western blot analysis and qRT-PCR revealed UBE2C down-regulation by the knockdown of FoxM1, whereas si-UBE2C did not affect FoxM1 expression in U87-MG cells; **p < 0.001. (B) Western blot analysis and qRT-PCR revealed that UBE2C was downregulated by FoxM1 knockdown, whereas si-UBE2C did not affect FoxM1 expression in U251 cells; **p < 0.001.

To investigate the effect of relatively high FoxM1 expression on UBE2C expression, we generated stable FoxM1-knockdown U87-MG and U251 cell lines, referred to as U87-shFoxM1 and U251-shFoxM1, respectively. Both cell lines showed significant FoxM1 inhibition on the mRNA and protein levels (left panels of Fig. 4A and B). Consequently, both UBE2C protein and mRNA expression levels were decreased in the stable FoxM1-knockdown cell lines. In addition, to further determine whether UBE2C promotes FoxM1 expression, we silenced UBE2C using siRNA in U87-MG and U251 cells and found that si-UBE2C failed to decrease FoxM1 expression (right panels of Fig. 4A and B). These data suggest that FoxM1 might transcriptionally regulate UBE2C expression.

### FoxM1 directly binds to and activates the UBE2C promoter

**Figure 5.**
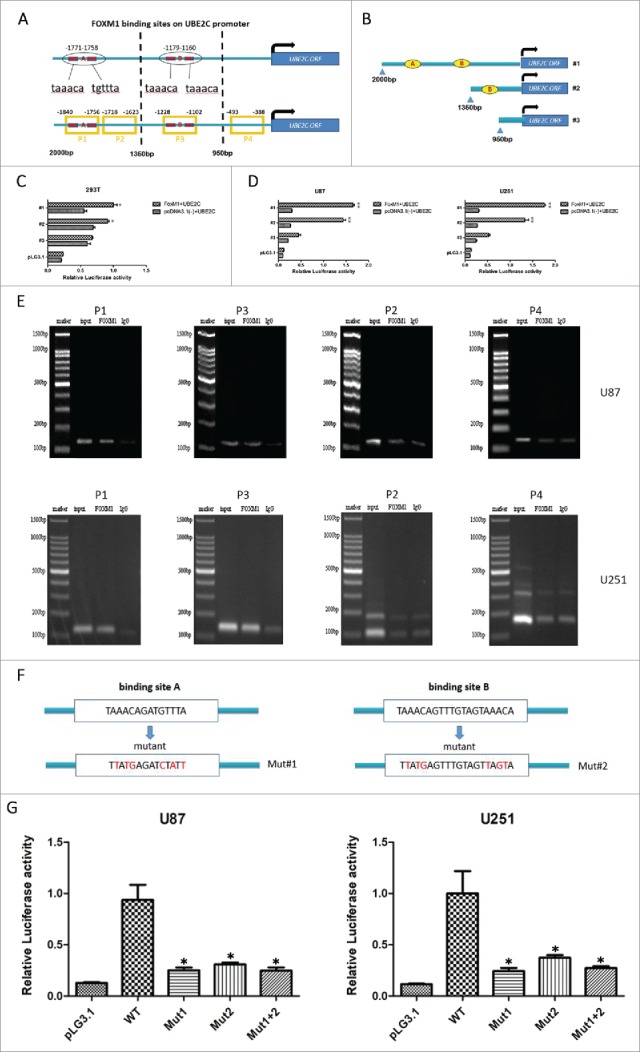
FoxM1 binds to and activates the UBE2C promoter**.** (A) The predicted sequences of putative FoxM1-binding sites in the −2000 bp region of the UBE2C promoter by gene sequence analysis. (B) The schematic representation of constructs harboring different fragments in the −2000 bp region of the UBE2C promoter. (C) The Dual Luciferase reporter assay showed the relative promoter activities of each construct in 293T cells; *p < 0.05, **p < 0.01. Each construct was cloned into the basic pGL3 firefly luciferase reporter vector. (D) The Dual Luciferase reporter assay showed that different reporter plasmids covering different regions of the UBE2C promoter mediated the transcriptional activities of FoxM1 in U87-MG and U251 cells. (E) The ChIP results clearly revealed the binding activities of FoxM1 on the UBE2C promoter region. In U87-MG and U251 cells, the amounts of promoter DNA that were associated after ChIP were quantitated by qRT-PCR using four pairs of primers (P1/P2 and P3/P4). IgG was used as a negative control. (F) Schematic representation of the FoxM1-binding sites; sequences are shown in both the wild-type and mutant forms. (G) Mutational analysis of the predicted FoxM1-binding sites with the wild-type and mutant reporter plasmids in U87-MG and U251 cells; *p < 0.001.

To explore whether UBE2C is a potential downstream target of FoxM1, the −2000 bp promoter region of the human UBE2C gene was searched for the FoxM1 consensus motif TAAACA and the palindrome TAAACA.{0,5}TGTTTA.^20^^,^^30^ We found two putative FoxM1-binding sites in the regions from −1771 to −1758 bp and −1179 to −1160 bp in the UBE2C promoter (Fig. 5A). To further confirm whether FoxM1 directly regulates UBE2C expression at the transcriptional level, three UBE2C promoter fragments covering the regions from −2000 (F1), −1350 (F2) and −950 (F3) to 0 bp were amplified and cloned into pGL3-basic luciferase plasmids (Fig. 5B). The promoter-luciferase plasmids were co-transfected into 293T cells with pcDNA3.1-FoxM1 or control plasmid vector pcDNA3.1, and promoter activities were measured. As shown in Fig. 5C, the F1 and F2 fragments induced remarkable transcriptional activities, whereas no obvious activity was found in response to the F3 fragment. Similar results were obtained in U87-MG and U251 cells (Fig. 5D). Our results indicate that FoxM1 is involved in the activity of the UBE2C promoter in the region from −2000 to −950 bp.

To further confirm the direct correlation between FoxM1 and the UBE2C promoter, we performed ChIP assays in U87-MG and U251 cells with four pairs of primers covering four regions of the UBE2C promoter: P1 (−1840 to −1756 bp), P2 (−1718 to −1623 bp), P3 (−1228 to −1102 bp) and P4 (−493 to −388 bp) (Table S2). As shown in Fig. 5E, the P1 and P3 primers yielded a higher amount of PCR product than the P2 and P4 primers in the two cell lines. Taken together, these findings suggest that FoxM1 transcriptionally regulates UBE2C expression by directly binding to the UBE2C promoter in glioma cells.

To assess the functional role of the putative FoxM1-binding sites in UBE2C transactivation, we induced several mutations within the region from −2000 to −950 bp of the UBE2C promoter using the pGL3-basic plasmid. As shown in Fig. 5F, predicted mutant binding sites contained mutation 1, mutation 2 and mutation 1 plus 2. Compared with the wild-type UBE2C promoter, mutations 1 or 2 resulted in a significant reduction in UBE2C promoter activity when co-transfected with the FoxM1 plasmid. Meanwhile, mutation 1 plus 2 also remarkably attenuated UBE2C promoter activity (Fig. 5G). Therefore, our results indicate that the two binding sites of FoxM1 are crucial for FoxM1 regulation of UBE2C promoter activation.

### Attenuation of UBE2C-induced autophagy in glioma cells

**Figure 6.**
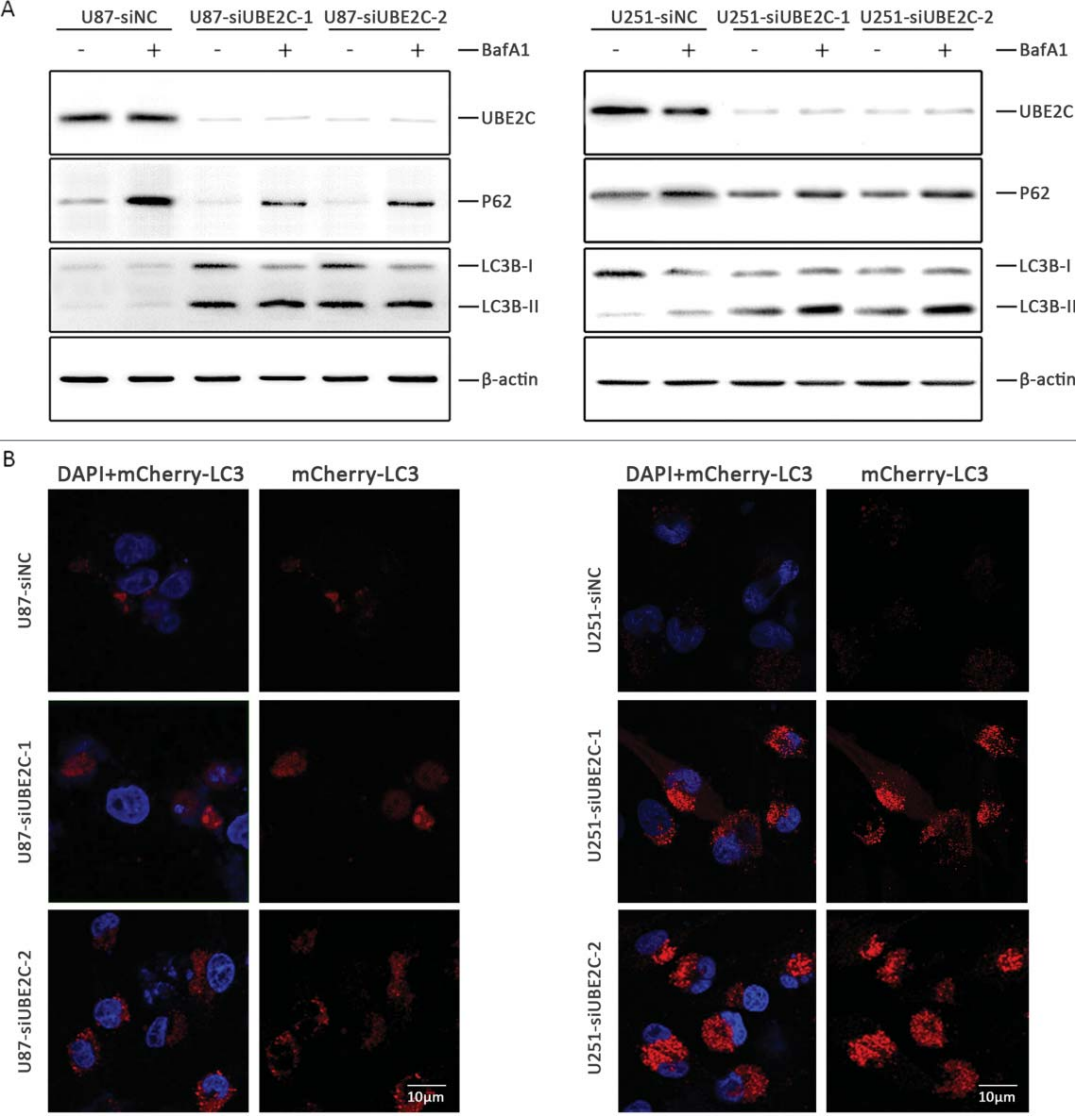
Autophagy induction by UBE2C attenuation. (A) U87-MG and U251 cells were transiently transfected with or without si-UBE2C for 48 h. Next, both cell lines were treated with the indicated concentrations of BafA1 along with the vehicle control; then, the expression of UBE2C, SQSTM1/p62 and LC3B-I/II was determined by Western blot. β-Actin was used as a loading control. (B) U87-MG and U251 cells were first transiently transfected with or without si-UBE2C for 48 h and were further transfected with an mCherry-LC3 vector for another 24 h. Next, autophagosomes were measured by confocal microscopy. Each image is a representation of 3 independent experiments performed in triplicate.

To demonstrate the corresponding function of UBE2C in the induction of autophagy in glioma cells, U87-MG and U251 cells were transiently transfected for 48 h with si-UBE2C or scrambled siRNA as a negative control (si-NC), respectively. Levels of LC3B-I (microtubule-associated protein 1 light chain 3; protein recruited from the cytosol), LC3B-II (protein recruited from autophagosomal membranes, where it is lipidated) and sequestosome 1 (SQSTM1/p62, a protein that links LC3 to specific ubiquitin substrates) were detected as an evaluation of autophagy.^32^ We found that UBE2C knockdown significantly increased the conversion of LC3B-I to LC3B-II and simultaneously triggered a decrease in SQSTM1 expression. Moreover, the effects of an additional treatment with bafilomycin A1 (BafA1, 10 nM), which disrupts autophagic flux by independently inhibiting acidification and autophagosome-lysosome fusion,^33^ were determined in both cell lines. Compared with BafA1 treatment alone, the combined treatment with si-UBE2C dramatically enhanced the conversion of LC3B-I to LC3B-II and the degradation of SQSTM1 (Fig. 6A), confirming that si-UBE2C indeed enhanced autophagic activity.

To further analyze the recruitment of LC3-II to autophagosomes in response to si-UBE2C treatment, cherry fluorescent protein-fused LC3B (mCherry-LC3) plasmids were transiently transfected into U87-MG (U87-LC3B) and U251 (U251-LC3B) cells, respectively. Thereafter, both cell types were treated with si-UBE2C or si-NC for 48 h. As shown in Fig. 6B, a remarkable accumulation of red puncta was observed in si-UBE2C-treated cells by confocal microscopy, whereas a diffuse and weak red signal was observed in si-NC-treated cells, suggesting that silencing UBE2C led to the activation of autophagy flux.

### Negative effect of UBE2C-attenuation on viability could be abrogated by BafA1

**Figure 7.**
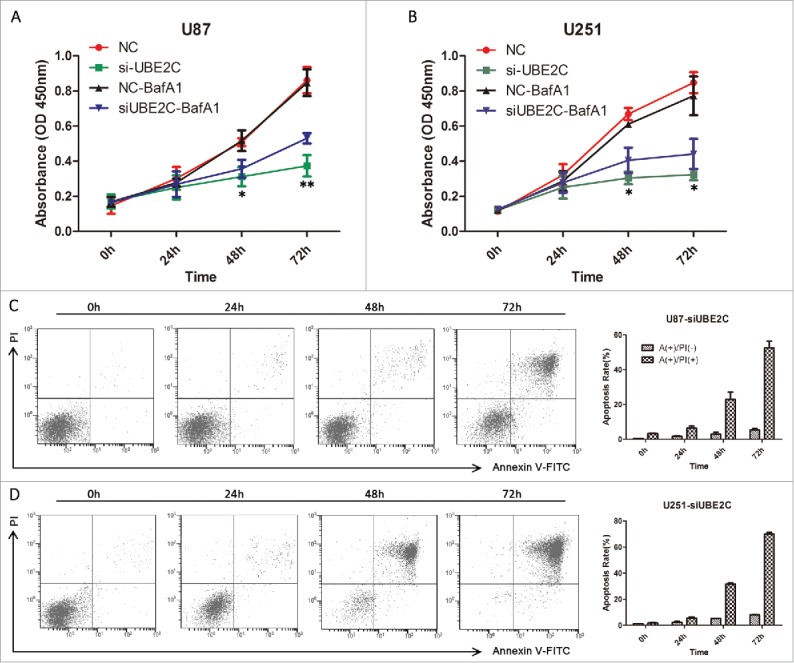
si-UBE2C inhibits cell viability, which can be abrogated by BafA1, but does not induce obvious apoptosis. U87-MG and U251 cells were first transiently transfected with si-NC or with si-UBE2C for 48 h (A-B) and were consequently treated with BafA1 at 10 nM for 1.5 h. Then, relative cell viabilities were measured using a CCK-8 assay at 24, 48 and 72 h. All CCK-8 assay results were obtained from three independent experiments, *p < 0.05. (C-D) In each panel, the lower left quadrant shows viable cells (Annexin V-FITC and PI negative), the lower right quadrant shows cells in early apoptosis (Annexin V-FITC positive and PI negative), the right upper panel shows cells that are at the end of apoptosis or are necrotic (Annexin V-FITC and PI positive), and the left upper panel shows damaged cells (Annexin V-FITC negative and PI positive). The percentages of cells in early and late apoptosis are shown in the histogram; *p < 0.05, **p < 0.01.

We assessed cell viability with or without being treated with the autophagy inhibitor BafA1 to assess the effects of autophagy on the negative effect of UBE2C attenuation on viability. First, U87-MG and U251 cells were transiently transfected with si-UBE2C and were subsequently treated with BafA1 (10 nM). Cell viability was then determined by a CCK-8 assay at 0, 24, 48 and 72 h after transfection. As shown in Fig. 7A, B, si-UBE2C obviously inhibited cell proliferative activities in a time-dependent manner compared with si-NC. Meanwhile, the combined si-UBE2C and BafA1 treatment also decreased cell proliferative activities compared with the si-NC treatment in a time-dependent manner, but the suppressive effect was significantly lower than those of the si-UBE2C and si-NC treatments (p < 0.05), suggesting that BafA1 exerted rescuing effects and that autophagy might participate in cell proliferation.

To further determine whether si-UBE2C-induced cell death is caused mainly by apoptosis, we assessed the percentage of apoptotic U87-MG and U251 cells using an Annexin V-FITC staining assay. After treatment with si-UBE2C for 24, 48 or 72 h, the proportion of PI-positive (i.e., dead) cells generally increased in a time-dependent manner (Fig. 7C, D) in both cell types. However, the proportion of Annexin V-positive (i.e., early apoptotic) cells in the si-UBE2C-treated group was very low. Therefore, the cell apoptosis rate was not significantly elevated when UBE2C was inhibited, which in turn suggested that autophagic cell death might be involved in this si-UBE2C-induced process.

### UBE2C knockdown inhibited the Akt-mTOR signaling pathway

**Figure 8.**
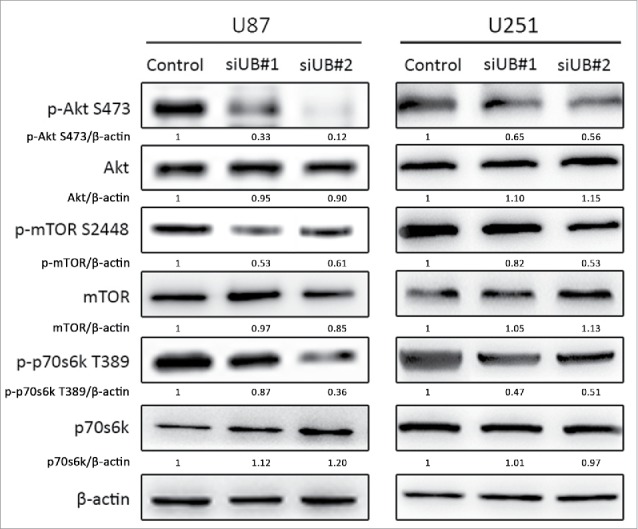
si-UBE2C inhibits the PI3K-Akt-mTOR signaling pathway in glioma cells. U87-MG and U251 cells were transiently transfected with si-UBE2C for 48 h, and the expression of phospho-Akt, phospho-mTOR and phospho-p70S6K1 was then analyzed by Western blot. β-Actin was used as a loading control.

Since the Akt-mTOR-p70s6k signaling pathway is known to be an important negative regulator of autophagy,^34^ we performed a Western blot analysis to evaluate the total and phosphorylated protein expression levels of the three key molecules in this pathway. As shown in Fig. 8, after treatment with si-UBE3C for 48 h, the phosphorylation levels of Akt, mTOR and p70s6k were significantly decreased in the si-UBE2C group compared with the control group, showing that si-UBE2C had ripple effects on the inactivation of the Akt-mTOR-p70s6k pathway. Collectively, these observations suggested that UBE2C attenuation might participate in the autophagic process through inhibiting the Akt-mTOR-p70s6k signaling pathway.

## Discussion

Our study focused on determining the molecular mechanism by which FoxM1 represents a pivotal transcriptional regulator of UBE2C, whose deregulation induces autophagic death in glioma cells. Previously, a series of in vitro and in vivo studies indicated that FoxM1 and UBE2C displayed high expression and activity levels in many cancer types, including glioma.^5-11^ In this study, we also found that UBE2C expression was strongly correlated with FoxM1 expression in gliomas. Moreover, high FoxM1 and UBE2C expression levels in gliomas were closely associated with a poor prognosis. A subsequent bioinformatics investigation predicted binding between the two molecules bind. Furthermore, we found that FoxM1 inhibition by transfection with FoxM1-shRNA significantly down-regulated UBE2C expression, whereas UBE2C knockdown failed to decrease FoxM1 expression. FoxM1 transactivated UBE2C by directly binding the UBE2C gene promoter. Therefore, exploring the potential mechanism by which FoxM1 acts on UBE2C may provide a novel therapeutic approach for treating gliomas.

The oncogenic transcriptional factor FoxM1 has been demonstrated to be a key regulator of the cell cycle, as it regulates the G1/S- and G2/M-phase transitions, as well as the progression to mitosis.^8^ In addition, many studies have shown the importance of FoxM1 in the tumorigenesis of several cancer types, and its overexpression is closely involved in tumor development and progression.^35-37^ The present study furthered our understanding of the mechanism by which FoxM1 regulates glioma oncogenesis in several ways. First, our IHC analyses showed a significant positive correlation between FoxM1 and UBE2C expression in 154 specimens. Simultaneously, a significant positive association between high FoxM1/UBE2C expression and poor prognosis was observed in the above 154 glioma cases, providing evidence that FoxM1 might regulate UBE2C expression. Second, to our knowledge, this is the first report to demonstrate that UBE2C is a direct target of FoxM1. In addition, we identified two FoxM1-binding sites, mapped between −1771 and −1758 bp and between −1179 and −1160 bp of the proximal UBE2C promoter. FoxM1 appeared to crucially regulate UBE2C expression through direct interaction with the UBE2C promoter, as mutations of the FoxM1-binding sites significantly down-regulated UBE2C promoter activity in GBM cells. Subsequently, our ChIP assay results further substantiated these findings. Collectively, our findings provide both clinical and mechanistic evidence that FoxM1 critically regulates UBE2C expression.

UBE2C is a key ubiquitin ligase and is involved in intracellular protein degradation via the mitotic spindle assembly checkpoint pathway.^21^ Notably, a variety of studies have revealed that UBE2C possesses a tumor stimulation function. As the ubiquitin-proteasome system is a complicated process that controls the continuous exchange of protein levels, aberrant protein degradation will result in cell death.^9^ Interestingly, most previous studies demonstrated that UBE2C overexpression was observed in several malignancies and was closely associated with patient prognosis. Donato et al.^26^ confirmed a clear correlation between UBE2C expression and the histological grade of astrocytic tumors. Jiang et al.^27^ analyzed the UBE2C expression status in astrocytomas of different grades and further indicated that UBE2C knockdown inhibited glioma cell proliferation. As the hallmarks of malignant tumors are characterized by abnormal cell growth and apoptosis, in our study, we performed CCK-8 and Annexin V staining assays to determine the effects of UBE2C deletion in U87-MG and U251 cells. Indeed, the data showed that cells transiently transfected with si-UBE2C for 48 h exhibited growth ability suppressed by approximately 40% compared with that of cells in the control group, which was consistent with previous reports. Interestingly, cells co-transfected with BafA1 and si-UBE2C for 48 h showed only a 20% reduction compared with the control. Furthermore, it is worth noting that the results of the Annexin V-FITC staining assay performed with U87-MG and U251 cells showed that the percentage of apoptotic cells accounted for only small amount of the overall cell death observed, indicating that UBE2C-induced cell death might be caused by a process other than apoptosis. Furthermore, it is worth noting that the results of the Annexin V-FITC staining assay performed with U87-MG and U251 cells showed that the percentage of early apoptotic cells accounted for only a small amount of the overall cell death observed, and no significant differences were observed over time; however, the proportion of cells at the late stage of apoptosis or in necrosis increased in a time-dependent manner. Thus, we speculated that a mechanism other than apoptosis resulted in glioma cell death. Based on this result and the results of the CCK-8 assay, we inferred that autophagic cell death might be involved in this process. As growing evidence has highlighted the importance of autophagy in anti-tumor therapies,^38-40^ we next investigated whether autophagy was induced and required for the attenuated UBE2C-mediated inhibition of cell proliferation.

Autophagy, a complex cellular degradation process, can exert a protective function under conditions of metabolic stress or nutrition starvation by digesting aged organelles with autophagy-specific lysosomes.^41^^,^^42^ However, recent studies have revealed that aggravated autophagy might suppress tumor progression by causing cell death as another type of programmed cell death aside from apoptosis, i.e., autophagic cell death.^43-45^ Compared with apoptosis, autophagic cell death has diverse features, including autophagosome aggregation, the conversion of LC3B-I to LC3B-II, and the attenuation of SQSTM1.^46^^,^^47^ In this study, we evaluated the effects of UBE2C on glioma cell autophagy, and we found that si-UBE2C triggered a significant increase in the conversion of LC3B-I to LC3B-II. LC3 is involved in the recruitment from the cytoplasmic LC3-I protein to the autophagosomal membrane, where it is lipidated (LC3-II).^48^ Simultaneously, si-UBE2C exerted a decrease in SQSTM1 expression, which is a protein that binds LC3 to ubiquitinated substrates; additionally, its degradation is normally considered an indicator of autophagy.^49^ To further confirm that si-UBE2C induces an increase in molecular markers of autophagy, U87-MG and U251 cells were transfected with an mCherry-LC3 vector, and autophagosomes were then measured by confocal microscopy.^50^ The results showed that si-UBE2C led to an increase in the number of red puncta. Moreover, co-treating the cells with si-UBE2C and the autophagy inhibitor BafA1, which suppresses the degradation of LC3-II, led to an additional accumulation of cherry-LC3 puncta. These findings indicated that si-UBE2C induced autophagy flux and that the promotive effects of UBE2C on glioma cells could be due to the inhibition of autophagy. In addition, since the PI3K-Akt-mTOR signaling pathway is known as a crucial negative regulator of autophagy, we detected the activation of Akt, mTOR and p70S6K in glioma cells. The data showed that the phosphorylation of these markers was markedly suppressed by silencing UBE2C, which might contribute to autophagic cell death in si-UBE2C-treated cells. However, as autophagy is a dynamic mechanism and influenced by complicated stimuli, further research on the effects and application of si-UBE2C in vivo is warranted.

In conclusion, our study provided the first evidence that FoxM1 regulated UBE2C expression via directly binding to the UBE2C promoter. Moreover, we demonstrated that the possible mechanism of the si-UBE2C-induced inhibition of glioma cell proliferation might be autophagic cell death induction and PI3K-Akt-mTOR signaling pathway inactivation. Our findings indicate a novel role of FoxM1 in the development of glioma and initially present an autophagy-related function of UBE2C, which may be a feasible target for molecular glioma therapies. Further investigations will be conducted to confirm the potential crosstalk among the FoxM1-UBE2C-meditated autophagy process and the intricate signaling cascades of the PI3K-Akt-mTOR pathway.

## Materials and methods

### Cell culture and glioma tissue samples

Human glioma cell lines U87-MG, U251, ln18 and U373 were obtained from the American Type Culture Collection (Manassas, VA, USA), and normal human astrocytes (NHAs) were purchased from Lonza. All cells were cultured in Dulbecco's modified eagle medium (DMEM) containing 10% fetal bovine serum at 37°C and 5% CO_2_.

A total of 154 glioma patients, including 32 grade I, 42 grade II, 45 grade III and 35 grade IV cases, were enrolled from January 2006 to July 2012 in the Department of Neurosurgery, The First Affiliated Hospital of Sun Yat-Sen University, China. Human glioma tissues were obtained from surgical specimens and immediately snap-frozen in liquid nitrogen until RNA and protein extraction. None of the patients had undergone chemotherapy or radiotherapy prior to surgery. In all, 27 paired normal tissues in non-functional areas served as the control group. All tissue samples were formalin-fixed, paraffin-embedded and assessed by two pathologists (LI Y. and HAN A.J) using the WHO classifications. The inclusion of patients in this study was unbiased and only dependent on the availability of tumor materials and clinical follow-up data. Clinical patient characteristics are provided in Table 1. All 154 patients were given a follow-up examination, and overall survival (OS) was measured from the day of the surgery until the last follow-up or death. The follow-ups were terminated in December 2015. This study was approved by the ethics committee of Sun Yat-Sen University, and written informed consent was obtained from all patients.

**Table 1.** Clinical characteristics of study samples.

				Gender	
WHO grade	Histological type	No. of patients	Mean age(yrs)	Male	Female
I	Pilocytic astrocytoma	32	16.97	18	14
	Choroid plexus papilloma				
II	Astrocytoma	42	30.95	23	19
	Oligodendroglioma				
III	Anaplastic astrocytoma	45	34.44	26	19
	Anaplastic Oligodendroglioma				
IV	GBM	35	44.43	19	16
Total		154	32.13	86	68

### Bioinformatics analysis

The NCBI database was searched to determine the chromosomal location in the genome. The UCSC Genome Browser (http://genome.ucsc.edu) was searched to investigate the bioinformatics of the UBE2C gene. The human UBE2C promoter sequence and the 2000-bp region upstream of it (UCSC version GRCh37/hg19) were searched.

### Reagents and antibodies

Anti-rabbit antibodies were obtained against FoxM1 (Santa Cruz, L3004), LC3B (MBL, PM036), sequestosome 1 (SQSTM1; MBL, PM045), Alexa Fluor 555 (Molecular Probes, C-22843), Akt (CST, 4685), phospho-Akt (CST, S473), mTOR (CST, 2983), phospho-mTOR (CST, S2448), p70s6k (CST, 2708), and phospho-p70s6k (CST, S424/T421). Anti-mouse antibodies were obtained against UBE2C (Abnova, H00011065-M01), β-actin (Abclone, AC004) and Alexa Fluor 488 (Molecular Probes, C-34775). The reagents used in this study included those of a Dual Luciferase Assay Kit (BioVision, USA) and the Lipofectamine™ 3000 reagent (Invitrogen, USA). UBE2C siRNA and FoxM1 shRNA were designed and synthesized by GenePharma (Shanghai, China). The UBE2C and mCherry-LC3 expression plasmids were purchased from Generay (Shanghai, China).

### Transient and stable transfection of glioma cells

To inhibit UBE2C expression, we transfected U87-MG and U251 cells with a UBE2C-siRNA oligonucleotide (50 nM) with the sequence AGUGGUCUGCCCUGUAUGAdTdT (si-1) or the sequence AGGGAUUUCUGCCUUCCCUdTdT (si-2) or with a control siRNA (50 nM). U87-MG and U251 cells were also transfected with a FoxM1-shRNA expression vector^14^ for stable transfection. Stably transfected cell lines were isolated by puromycin selection. To avoid clonal selection, we pooled all of the puromycin-resistant colonies to establish stable transfectants.

### Immunohistochemical staining and scoring

Glioma and normal brain tissue samples were cut into 5-μm-thick sections and mounted on 3-aminopropyltriethoxysilane-coated glass slides. The sections were de-waxed, rehydrated and treated with 1X antigen retrieval solution (Beyotime). After endogenous peroxidase blocking (3% H_2_O_2_), the slides were incubated in monoclonal mouse anti-human UBE2C antibody (1:50 dilution), and in polyclonal rabbit anti-human FoxM1 antibody (1:100 dilution), respectively, overnight at 4°C. Then, the slides were sequentially processed using anti-mouse/rabbit biotinylated antibody (Dako) for 30 min at room temperature, followed by development with diaminobenzidine for visualization. All sections were counterstained with Mayer's hematoxylin. The tissue sections were washed with 1X phosphate-buffered saline (PBS) at each immunostaining step and then dehydrated and mounted.

The following evaluation was based on both the proportion of positively stained tumor cells and the staining intensity. The immunoreactive score (IRS) of glioma cells was classified as 0 (<5%), 1 (5–25%), 2 (26–50%), or 3 (>50%). The staining intensity was visually scored and grouped as follows: 0 (negative), 1 (weak), 2 (moderate), or 3 (strong). The IRS was obtained by multiplying the percentage and the intensity score of each case. Samples with a total IRS of <4 were determined as having low UBE2C or FoxM1 expression, and samples with a total IRS of ≧4 were classified as having high expression.

### Immunofluorescence microscopy

Paraffin-embedded, 5-μm-thick sections of the gliomas with different histological grades were deparaffinized, heated in citrate buffer (pH 6.0) for epitope retrieval, and then blocked with PBS containing 5.0% bovine serum albumin (BSA). The sections were incubated with primary rabbit anti-FoxM1 (1:25 dilution) and mouse anti-UBE2C (1:15 dilution) antibodies at 4°C overnight. Subsequently, the sections were incubated with goat anti-rabbit secondary antibody conjugated to Alexa Fluor 555, which fluoresces red, and anti-mouse secondary antibody conjugated to Alexa Fluor 488, which fluoresces green, at 37°C; then, nuclei were counterstained with 4′, 6-diamidino-2-phenylindole. Images were captured using an inverted fluorescence microscope (Leica, DMI4000B).

### Quantitative reverse transcription polymerase chain reaction (qRT-PCR)

Total RNA was extracted from tissues and cell lines using TRIzol reagent (Invitrogen, USA), and 2 µg of total DNA-free RNA was used to synthesize cDNA using a ReverTra Ace qPCR RT Kit (Toyobo, Japan) according to the manufacturer's instructions. The reactions were carried out in 96 well plates using 1 µl of cDNA with Thunderbird SYBR qPCR Mix (Toyobo, Japan), to which gene-specific forward and reverse PCR primers were added. qRT-PCR was performed to detect UBE2C or FoxM1 mRNA expression. The conditions used for PCR were 1 cycle of 10 min at 95°C, and then 40 cycles of 10 sec at 95°C, followed by 40 cycles of 34 sec at 55°C. Each reaction was carried out in triplicate. Real-time PCR primers were designed using Primer Express v 2.0 software (Applied Biosystems), and the primer sequences used were as follows: UBE2C-F: 5′- TGATGTCTGGCGATAAAGGGA-3′, UBE2C-R: 5′- ATAGCAGGGCGTGAGGAAC-3′; FoxM1-F: 5′-ACGTCCCCAAGCCAGGCTC-3′, FoxM1-F: 5′-CTACTGTAGCTCAGGAATAA-3′.

### Western blot analysis

Total protein of tissue and cell lysates was prepared in RIPA lysis buffer (Beyotime, China). Protein concentrations were measured using a bicinchoninic acid protein assay kit (Pierce Biotechnology, Inc., USA). Equal amounts of protein lysates were electrophoretically separated by 12% sodium dodecyl sulfate-polyacrylamide gel electrophoresis and transferred to polyvinylidene difluoride (PVDF) membranes (Invitrogen, Carlsbad, CA). The PVDF membranes were blocked with 1% BSA in Tris-buffered saline (TBS)/0.1% Tween for 2 h at room temperature and then incubated with the appropriate primary antibodies overnight at 4°C. After 3 10-min washes in TBS/0.1% Tween, the membranes were incubated with the corresponding secondary antibodies for 1 h at room temperature, followed by 3 10-min washes in TBS/0.1% Tween. Then, protein visualization was accomplished with an automatic chemiluminescence imaging analysis system (Tenon 5200, China).

### Promoter constructs, dual luciferase reporter assay and ChIP assay

The UBE2C promoter and truncated promoters with different lengths (described previously) were cloned into a pGL3-basic vector (Promega). The UBE2C mutant promoter constructs were generated using a QuikChange Site-Directed Mutagenesis Kit (Generay, Shanghai). The luciferase activities were detected with a Dual Luciferase Assay (Promega, USA) according to the manufacturer's instructions.

For the dual-luciferase assays, Lipofectamine2000 (Invitrogen) was used to co-transfect 293T or glioma cells with 2 μg of either FoxM1-Flag or control pGL3.1 vectors and 2 μg of luciferase reporter constructs containing different UBE2C promoter regions. The cells were cultured for 48 h after transfection and lysed in the culture dishes with lysis buffer. The relative luciferase activity was determined by an Infinite F500 Luminometer (Tecan, Switzerland), and the transfection efficiency was normalized by Renilla activity. All experiments were performed at least twice in triplicate.

Chromatin immunoprecipitation (ChIP) assays were performed. A total of 1 mL of anti-FoxM1 antibody and control IgG was used. Immunoprecipitated DNA was precipitated with ethanol and resuspended in 10 mL of double-distilled water. The total input samples were resuspended in 100 mL of double-distilled water and diluted 1:200 prior to PCR. The purified ChIP DNA sample and total input DNA sample were subjected to PCR and qRT-PCR analyses using the primers listed in Table S2. The PCR products were separated on 2.5% agarose gels and analyzed through ethidium bromide staining. All ChIP assays were conducted at least three times.

### Analysis of cell viability and apoptosis

A Cell Counting Kit 8 (CCK-8) assay was performed to determine cell proliferation. In brief, U87-MG and U251 cells were cultured in 96-well plates at a density of 1 ×  10^5^ cells per well for 24 h. Each group was designed to have 5 replicate wells. After transfection with si-UBE2C for 48 h, the supernatant was removed, and 100 μL of DMEM was added in the presence of 10 μL of CCK-8 reagent (Thermo, USA) at 24, 48, 72 or 96 h. The absorbance of the medium at 450 nm was detected after 2.5 h of incubation at 37°C.

To determine the apoptosis rate, a FITC Annexin V Apoptosis Detection Kit (BD Pharmingen, USA) was used in accordance with the manufacturer's instruction and was analyzed by flow cytometry (BD Bioscience, USA).

### Statistical analysis

Statistical calculations were performed using SPSS software (version 20.0, SPSS Inc., Chicago, IL, USA) and GraphPad Prism v5.0 (GraphPad Software, Inc., La Jolla, CA, USA). The χ^2^ test was used to compare the different variables, the Kaplan–Meier method was used to evaluate survival rate, and the prognosis was analyzed by univariate and multivariate Cox proportional hazard regression models; p < 0.05 was considered significant.

## Supplementary Material

Supplemental Files
