# Supplementary material for: Forkhead Box M1 positively regulates UBE2C and protects glioma cells from autophagic death
Source: Cell Cycle. 2017 Aug 2;16(18):1705–18. doi: 10.1080/15384101.2017.1356507 (PMC5602297; doi:10.1080/15384101.2017.1356507)
Supplement: Supplemental Files [file kccy-16-18-1356507-s001.zip › Supplemental Table 2.docx]

Sup Tab 2 Information of the primers for ChIP

| **Primer** | **Forward Sequence** | **Reverse Sequence** | **Location** | **TSS** |
| --- | --- | --- | --- | --- |
| P1 | ATGACCACTCTCCCTTGCTC | CTGTAATCATCACTGGCCGC | chr20:44439495+44439579 | 1756-1840 |
| P2 | TGCCTCCAAATAGCCCCTAG | TCAGATCCAGGCATCCTCTG | chr20:44439617+44439712 | 1623-1718 |
| P3 | AAGGGCTCTAGAAGTGGGTG | AAATGGGCAAAGGGAAGACG | chr20:44440107+44440233 | 1102-1228 |
| P4 | CTTAGGACGCAGCAAAGGTG | GCCCAGCCTTGATCCAATTT | chr20:44440842+44440947 | 388-493 |
