# Supplementary material for: Forkhead Box M1 positively regulates UBE2C and protects glioma cells from autophagic death
Source: Cell Cycle. 2017 Aug 2;16(18):1705–18. doi: 10.1080/15384101.2017.1356507 (PMC5602297; doi:10.1080/15384101.2017.1356507)
Supplement: Supplemental Files [file kccy-16-18-1356507-s001.zip › Supplemental Table 1.docx]

| **Sup Table 1** |  |  |  |  |  |
| --- | --- | --- | --- | --- | --- |
| **Association between UBE2C expression and different clinicopathological features of gliomas** | | | | | |
|  |  | UBE2C expression | |  |  |
| Clinical Parameter | n | (Low=57) | (High=97) | *X^2^* | p-value |
| Gender |  |  |  |  |  |
| Male | 86 | 31 | 55 | 0.078 | 0.867 |
| Female | 68 | 26 | 42 |  |  |
| Age |  |  |  |  |  |
| ≤30 | 74 | 43 | 31 | 28.042 | ＜0.001 |
| 30-50 | 52 | 11 | 41 |  |  |
| ＞50 | 28 | 3 | 25 |  |  |
| Preoperative Epilepsy |  |  |  |  |  |
| Yes | 36 | 16 | 20 | 1.113 | 0.327 |
| No | 118 | 41 | 77 |  |  |
| KPS |  |  |  |  |  |
| ≥80 | 90 | 33 | 57 | 3.996 | 0.066 |
| ＜80 | 64 | 24 | 40 |  |  |
| Duration of Symptoms |  |  |  |  |  |
| ≤3m | 99 | 33 | 66 | 2.332 | 0.312 |
| 3-6m | 52 | 22 | 30 |  |  |
| ＞6m | 3 | 2 | 1 |  |  |
| Stage |  |  |  |  |  |
| LGG(WHO I&II) | 74 | 39 | 35 | 15.041 | ＜0.001 |
| HGG(WHO III&IV) | 80 | 18 | 62 |  |  |

 Comparisons within groups were made by using the Chi-squared test. A p-value of less than 0.05 was considered statistically significant. Abbreviations: KPS=Karnofsky Performance Status, WHO=World Health Organization.
